# Evolution of Sex Chromosomes: The Case of the White Campion

**DOI:** 10.1371/journal.pbio.0030028

**Published:** 2004-12-21

There are many different sex-determining systems in plants and animals with separate sexes (dioecious species). In some species, environmental factors activate sex-determining genes that trigger expression of genes leading to male or female development. Other species have evolved specialized sex chromosomes. In the well-known X-Y system of mammals, individuals inheriting a Y chromosome become males, and XX individuals become females.

Sex chromosomes have arisen independently in many taxonomic groups. It is an interesting question whether the same mechanisms were involved each time. Similarities in sex chromosome evolution have been reported between birds and mammals (although in birds, females are the heterozygous sex). In a new study, Michael Nicolas and colleagues uncover striking parallels in the details of sex chromosome evolution between mammals and a far more distant group: plants.

Sex chromosomes are an oddity in flowering plants. They are limited to dioecious species, a subset of plants that carry male and female organs (stamens and carpels, respectively) on separate individuals (most flowering plants are hermaphrodites). The genus Silene, which includes the White Campion, includes both dioecious and hermaphrodite species. The authors focus on three dioecious species, Silene dioica, S. latifolia, and S. diclinis, which share an X-Y sex-determination system where Y specifies maleness.

The theory of sex chromosome evolution holds that sex chromosomes were once homologs (a pair of equivalent autosomes—the non-sex chromosomes) that evolved different morphology and gene content because they lost their ability to recombine. Suppression of recombination is thought to start around the sex-determining region, but may eventually affect much of the sex chromosomes. Recombination is a key genetic process in which two chromosomes pair and exchange their sequences. In the absence of recombination, the two chromosomes of a pair evolve separately.[Fig pbio-0030028-g001]

**Figure pbio-0030028-g001:**
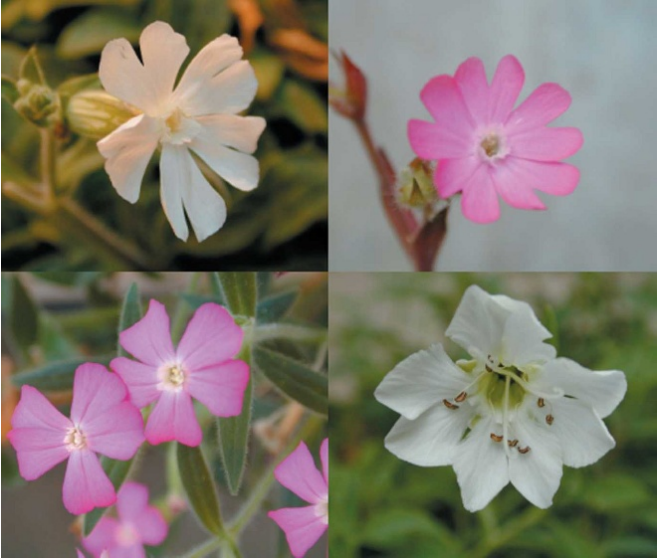
Flowers of Silene species, clockwise from top left: male flowers of S. latifolia and S. dioica, hermaphrodite flower of S. vulgaris, male flower of S. diclinis

In the case of mammals, whose sex chromosomes evolved about 320 million years ago, loss of recombination led to widely diverged X and Y chromosomes that pair only over a very small region, the pseudoautosomal region (PAR; because in this region the X and Y still behave like autosomes). The X and Y chromosomes of dioecious Silene species are morphologically distinct, like those of mammals, and they also have a PAR and a nonrecombining region. Nicolas and colleagues' results shed some light on how recombination suppression evolved on the Silene sex chromosomes.

The authors studied four genes outside the PAR on the Silene X chromosomes that are also present on their Y chromosomes. They mapped the genes relative to the PAR and compared the nucleotide sequences of the X and Y version of each gene in each species. As expected of sequences that no longer recombine, the X and Y versions of each gene have diverged. Strikingly, the extent of nucleotide divergence increases with the gene's distance from the PAR.

Evolutionary biologists use sequence divergence as a clock: the longer two originally identical sequences have been isolated from one another, the more independent mutations they accumulate. The picture that emerges from the Silene data is one of a progressive suppression of recombination, gradually diminishing the PAR. A similar scenario has been proposed in mammals and birds. However, the authors estimate that the Silene sex chromosomes started diverging only 10 million years ago. The Silene chromosomes might therefore offer a better chance to observe recombination suppression in its early stages, and perhaps to get at its mechanisms.

The authors also report evidence for some degeneration of the Silene Y chromosome genes. Y degeneration is well documented in mammals, in which most X-linked genes have no Y-linked counterparts. Understanding X-Y divergence in Silene species may thus shed light on the evolution of sex chromosomes in vertebrates as well.

